# Enhancing hair regeneration: Recent progress in tailoring nanostructured lipid carriers through surface modification strategies

**DOI:** 10.5599/admet.2376

**Published:** 2024-07-20

**Authors:** Omar M. Atrooz, Nasim Reihani, M. R. Mozafari, Ahmad Salawi, Elham Taghavi

**Affiliations:** 1 Department of Medical Laboratory Sciences, Faculty of Applied Medical Sciences, Al-Ahliyya Amman University, Amman 19328, Jordan; 2 Department of Biological Sciences, Mutah University, Mutah, Jordan; 3 Australasian Nanoscience and Nanotechnology Initiative (ANNI), Monash University LPO, Clayton, Victoria 3800, Australia; 4 Department of Pharmaceutics, College of Pharmacy, Jazan University, Jazan, 45142, Saudi Arabia; 5 Faculty of Fisheries and Food Science, Universiti Malaysia Terengganu, 21030 Kuala Nerus, Terengganu, Malaysia

**Keywords:** Nanostructured lipids, hair growth, surface modifications, nanotechnology

## Abstract

**Background and purpose:**

Hair loss is a prevalent problem affecting millions of people worldwide, necessitating innovative and efficient regrowth approaches. Nanostructured lipid carriers (NLCs) have become a hopeful option for transporting bioactive substances to hair follicles because of their compatibility with the body and capability to improve drug absorption.

**Review approach:**

Recently, surface modification techniques have been used to enhance hair regeneration by improving the customization of NLCs. These techniques involve applying polymers, incorporating targeting molecules, and modifying the surface charge.

**Key results:**

The conversation focuses on how these techniques enhance stability, compatibility with the body, and precise delivery to hair follicles within NLCs. Moreover, it explains how surface-modified NLCs can improve the bioavailability of hair growth-promoting agents like minoxidil and finasteride. Furthermore, information on how surface-modified NLCs interact with hair follicles is given, uncovering their possible uses in treating hair loss conditions.

**Conclusion:**

This review discusses the potential of altering the surface of NLCs to customize them for enhanced hair growth. It offers important information for upcoming studies on hair growth.

## Introduction

**Figure 1.**
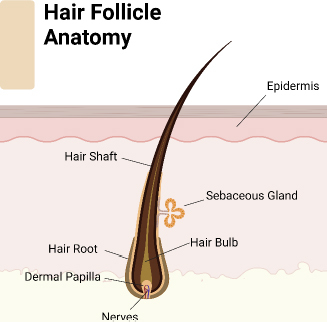
Schematic representative of hair follicle anatomy.

Hair regeneration remains a pivotal concern in clinical and cosmetic dermatology, driven by the psychological and social impact of hair loss on individuals [1]. Manipulated or left-natural hairstyles have a vital impact on shaping the face and highlighting facial characteristics, substantially contributing to one’s visual appearance [2]. Multiple research studies have demonstrated the psychological impacts of hair loss, particularly in cases of androgenetic alopecia (male and female pattern baldness) [3]. Individuals experiencing hair loss or thinning hair sometimes experience humiliation, social discomfort, and reduced self-assurance [4]. This emotional anguish can extend beyond individual consciousness and affect interpersonal relationships, professional growth, and general state of being. One must understand the importance of the physiological principles of hair growth [5]. Hair follicles (Figure 1) go through cyclical phases of development, regression, and dormancy. Complex molecular signaling networks control these phases, including growth factors, hormones, and cytokines. Disruptions in these processes can lead to hair loss or hindered regrowth, which can be attributed to genetic predisposition, hormone imbalances, or environmental causes [6]. Studying the scientific causes of hair growth provides insights into hair-related problems and drives progress in the field. To summarize, hair growth holds great importance beyond its visual attractiveness, including psychological, cultural, and physiological aspects. Recognizing the connection between hair and self-esteem highlights the importance of implementing thorough strategies to address hair concerns, ensuring overall well-being, and promoting a positive body image.

Treating hair loss can be challenging as it involves a complex interplay of genetic, hormonal, environmental, and physiological factors. Despite the progress made in medical technology, tackling hair loss continues to be challenging, marked by several significant obstacles as follows [7]:

1. Varied causes of hair loss can arise from a multitude of circumstances, including genetic predisposition (such as androgenetic alopecia), hormonal imbalances (such as thyroid problems), autoimmune conditions (such as alopecia areata), stress as an effect of environment, and infections of the scalp. Diagnosing and treating each cause requires a unique approach, which adds complexity to the treatment process [8].
2. Several medications and procedures can help with hair loss, but their effectiveness may vary depending on the root cause and individual reaction. Established treatments such as minoxidil and finasteride have demonstrated efficacy in treating certain types of hair loss. However, their effectiveness may vary among individual patients. In addition, techniques such as hair transplants yield durable results but may be intrusive and inappropriate for certain persons [7,9].
3. Limited comprehension of the biology of hair despite significant progress in elucidating the molecular mechanisms behind hair development and loss, our knowledge still needs to be completed. Several aspects of hair follicle biology, such as controlling follicle cycling, cell communication, and reaction to external stimuli, are currently under study. The little understanding hinders advancements in developing targeted therapies and predicting treatment strategies [10].
4. Transporting medication effectively to the hair follicle can be challenging due to the complexity of delivering the medicinal chemicals [7]. For topical products to be effective, they must penetrate the scalp barrier and reach the hair follicle with sufficient strength to achieve desired outcomes. Systemic medications may face challenges like metabolism, distribution, and unexpected side effects, reducing their effectiveness and tolerability [11].
5. Psychological effects and patient anticipation of the emotional well-being of individuals can be significantly affected by hair loss, leading patients to have optimistic expectations for the outcome of their therapy [12]. Striking a balance between these expectations and providing realistic predictions can be challenging, particularly due to individuals’ varying responses to therapy. Failing to meet expectations can worsen mental distress and dissatisfaction with the results of treatment [9,10].
6. Sustaining effectiveness and adherence over an extended period of numerous hair loss treatments necessitate ongoing adherence to ensure continued effectiveness, which might present difficulties in patient compliance. Adherence rates to therapy can be influenced by cost, convenience, and side effects, impacting treatment outcomes over time. Moreover, ceasing the treatment could result in a relapse or exacerbation of hair loss, highlighting the significance of continuous therapy [7]. To address these problems, we need a comprehensive approach that integrates molecular biology, pharmacology, and advancements in clinical practice. Collaboration among researchers, physicians, and industry stakeholders is essential to address these challenges and develop better and more accessible treatments for hair loss conditions [12].

**Figure 2.**
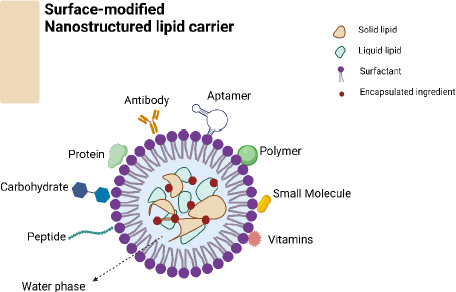
Different surface modification of nanostructured lipid carriers (NLCs).

Surface-altered nanostructured lipid carriers (NLCs) (Figure 2) provide a promising approach to enhancing hair growth by utilizing nanotechnology to address the constraints of traditional formulations. NLCs, or nanostructured lipid carriers, are composed of solid and liquid lipids, resulting in advantages such as higher drug loading capacity, controlled release kinetics, and improved stability [13]. Surface-functionalized NLCs can be customized to target hair follicles by incorporating specific ligands or bioactive compounds. This customization enhances the treatment’s effectiveness while minimizing potential negative effects [11]. Surface-altered NLCs show important characteristics that suggest they could improve hair growth treatment. A larger surface area compared to volume and small particle size help drugs penetrate hair follicles effectively and deliver bioactive substances to specific targets. Surface modification additionally improves the targeting of follicles, guaranteeing the best possible deposition of drugs inside the microenvironment of the follicles [11,14]. NLCs offer a controlled release of bioactive substances by enclosing them within a lipid matrix; this allows prolonged medication retention at the target site and reduces the dosing frequency. Surface modification helps adjust release patterns precisely, resulting in prolonged therapeutic effects while reducing overall exposure and adverse reactions [15].

NLCs can be modified on their surface by targeting ligands, such as peptides or antibodies, allowing them to bind specifically to receptors in the cells of the hair follicle epithelium [16]. This interaction enhances the absorption and internalization of NLCs by the hair follicles, expanding the range of available medications and promoting specific therapeutic effects [11,17]. The lipid-based structure of NLCs provides natural protection for enclosed bioactive substances, shielding them from degradation and enzymatic metabolism. The stability of NLCs is enhanced by surface modification, which helps protect them from being cleared too soon and supports their longer circulation in the skin environment [18]. Combination therapy with NLCs enables the encapsulation of multiple bioactive compounds with complementary modes of action; this promotes hair growth by enabling synergistic effects. Surface modification allows for precise control of the timing and location of medication release, improving positive interactions within the hair follicle environment [19]. Surface modification methods can be customized to address specific hair growth issues or individual patient characteristics, allowing for personalized treatment strategies. This customization improves the effectiveness of treatment by refining the parameters of medication distribution and addressing the individual differences in the response of hair follicles [20]. Surface-activated NLCs have the potential to enhance hair growth by delivering bioactive compounds directly to the hair follicles in an effective and long-lasting manner. Further research is needed to fully understand the most effective methods for developing treatments for hair loss using NLC; this includes ascertaining the appropriate composition, understanding their mechanisms of action within the body, and evaluating their efficacy in clinical environments [21].

This review paper aims to thoroughly investigate recent advancements in hair regeneration, specifically concentrating on the novel method of customizing NLCs through surface modification techniques. The article aims to explain how various surface modification techniques can improve NLCs to enhance the delivery of bioactive compounds to hair follicles. In addition, the goal is to provide a deep understanding of the processes that control the interaction between surface-modified NLCs and hair follicles, highlighting their potential in treating hair loss conditions.

## Overview of hair growth and hair loss

**Figure 3.**
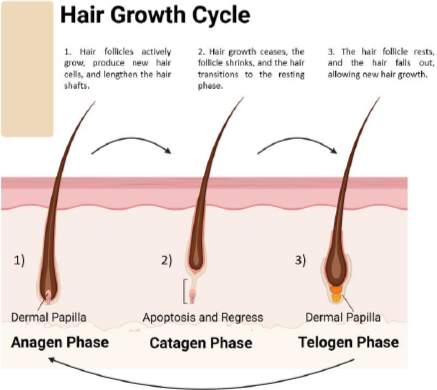
Hair growth cycle stages.

The hair follicle cycle (Figure 3) is a dynamic process involving growth, regression, and rest stages. This repetitive cycle controls the constant regeneration of hair follicles and influences hair size and thickness. The hair growth process consists of three primary stages [22]: 1. Anagen phase (growth phase), the active growth phase of the hair follicle; during this process, hair cells divide rapidly, and new hair shafts are formed. This phase typically lasts 2 to 7 years and is influenced by genetic and environmental factors and the hair follicle’s location on the body. The anagen phase’s duration mainly decides the hair shaft’s maximum length. Hair follicles in this phase are actively nourished by blood vessels, resulting in rapid hair growth. 2. Catagen phase (transitional phase) represents a transitional period between the growth and resting phases of the hair follicle. During this phase, the hair follicle undergoes apoptosis, which results in the cessation of hair growth. The catagen phase is relatively short and lasts approximately 2 to 3 weeks. As the hair follicle regresses, the lower portion detaches from the dermal papilla, signaling the end of active hair growth. 3. During the telogen phase, also known as the resting phase, the hair follicle is inactive and disconnected from the dermal papilla. Hair growth stops, and the hair shaft remains anchored in the follicle without actively growing. This phase lasts approximately 3 to 4 months. Afterward, the hair follicle re-enters the growth phase to start a new hair growth cycle [10,22-24]. Around 10 to 15 % of hair follicles on the scalp are in the telogen phase [25].

Following the telogen phase, the hair follicle re-enters the anagen phase, beginning a new hair growth cycle. The duration of each phase and the overall hair growth cycle can vary depending on individual factors such as genetics, age, hormonal fluctuations, and environmental influences. The synchronized progression through these phases ensures that hair follicles are continuously renewed and regenerated, maintaining the integrity and functionality of the hair. Understanding the hair growth cycle is crucial for clarifying the causes of hair disorders and creating specific treatments for hair loss conditions [25,26].

## Hair loss causes and types, current treatments, and their limitations

Hair loss, or alopecia, can result from various underlying causes, from genetic predisposition to environmental factors and medical conditions. Understanding the common causes and types of hair loss is essential for accurate diagnosis and targeted treatment. Two prevalent types of hair loss are androgenic alopecia and telogen effluvium [8].

Androgenic alopecia, also known as male-pattern or female-pattern baldness, is the predominant type of hair loss in both males and females. It is characterized by a gradual and predictable loss of hair, primarily from the front and top areas of the scalp in men and widespread thinning of hair across the top of the head in women. Androgenic alopecia is a condition determined by genetics and affected by androgen hormones, specifically dihydrotestosterone (DHT) [27,28]. This hormone causes hair follicles to shrink over time, leading to shorter and thinner hair. Eventually, the follicles become inactive. Androgenic alopecia in men is characterized by a receding hairline and thinning crown, whereas women may experience diffuse thinning without specific patterns. Androgenic alopecia can be treated in various ways, including applying minoxidil directly to the scalp, taking finasteride orally to reduce DHT production, undergoing hair transplantation, or receiving low-level laser therapy [4,29].

Telogen effluvium is a type of hair loss that does not cause permanent scarring. It is characterized by a sudden interruption of the hair development cycle, leading to significant hair shedding. This condition can be triggered by various factors, such as physical stressors like childbirth, surgery, severe illness, or emotional stress; hormonal changes like those during pregnancy or menopause; lack of essential nutrients; medications; and underlying medical issues such as thyroid disorders or autoimmune diseases. Telogen effluvium is usually characterized by widespread hair loss 2 to 3 months after the triggering event, which happens when the afflicted hair follicles enter the resting phase, known as telogen, prematurely and shed the hair shafts [30,31].

In contrast to androgenic alopecia, hair loss in telogen effluvium can be reversed, and hair will regrow once the underlying cause is treated. Treating telogen effluvium involves identifying and addressing the underlying cause, including correcting nutritional deficiencies, adjusting medication schedules, or practicing stress management techniques. In addition, implementing supportive measures such as adopting gentle hair care techniques and making nutritional alterations can facilitate hair restoration. To accurately diagnose and create personalized treatment plans, it is crucial to understand the specific characteristics and underlying causes of androgenic alopecia and telogen effluvium. Although androgenic alopecia usually needs continuous treatment to slow the progression and encourage hair growth, telogen effluvium usually improves with correct intervention for the root cause [32].

Current treatment options (Table 1) for hair loss encompass a variety of approaches, including medications, surgical procedures, and non-invasive therapies [33]. While these treatments can be effective to different extents, each method has limitations and factors to consider.

**Table 1.** Currently available treatment drugs for hair growth.

CAS number	Solubility	Formula	Chemical structure	Common ame	Ref.
38304-91-5	Hydrophilic	C_9_H_15_N5O	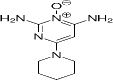	Minoxidil	[44]
98319-26-7	Hydrophobic	C_23_H_36_N_2_O_2_	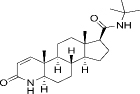	Finasteride	[45]
164656-23-9	Hydrophobic	C_27_H_30_F_6_N_2_O_2_	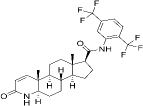	Dutasteride	[46]
50-23-7	Hydrophobic	Cortisol (C_21_H_30_O_5_)	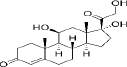	Corticosteroids	[47]
50-22-6	Hydrophobic	Corticosterone (C_21_H_30_O_4_)	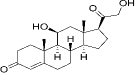		
53-06-5	Hydrophobic	Cortisone (C_21_H_28_O_5_)	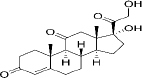		
52-39-1	Hydrophobic	aldosterone (C_21_H_28_O_5_)	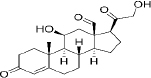		

### Medications

Topical Minoxidil, available in various strengths without a prescription, is widely used for treating hair loss. It stimulates hair growth by extending the anagen phase, enlarging the follicles, and improving blood circulation to the scalp [34]. Nevertheless, the efficacy of minoxidil varies from person to person, and consistent usage is necessary to sustain the desired outcomes. Typical adverse effects include scalp inflammation and undesired facial hair growth [35].

Oral finasteride, a 5-alpha reductase inhibitor, works by blocking the conversion of testosterone to DHT, thereby reducing DHT levels in the scalp and slowing hair loss in men with androgenic alopecia. It is not approved for use in women due to potential teratogenic effects. Side effects may include sexual dysfunction and mood changes [36,37]. Like finasteride, dutasteride inhibits 5-alpha reductase but is not approved by regulatory agencies in many countries for hair loss treatment. It may be considered off-label in some cases [38].

Corticosteroid solutions (*e.g.* topical corticosteroids) or foams can reduce inflammation and suppress immune responses in conditions such as alopecia areata. However, long-term use can lead to skin atrophy and other adverse effects [39,40].

### Surgical procedures

Follicular unit transplantation (FUT) and follicular unit extraction (FUE) are commonly used techniques for hair transplants. These techniques involve moving healthy hair follicles from a donor location to places where hair is thinning or lost. Although hair transplantation offers long-lasting outcomes, it is a surgical procedure that necessitates a thorough evaluation of the availability of donor hair, scalp flexibility, and aesthetic goals. Furthermore, the care and recovery period after a surgical procedure can be extended [41,42].

Scalp reduction which is a surgical procedure involves excising the bald scalp and stretching the hair-bearing scalp to cover the area. It is less commonly performed today due to the advent of hair transplantation techniques [43].

### Non-invasive therapies

Low-level laser therapy (LLLT) devices emit low-energy laser light to stimulate hair follicles and promote hair growth. While LLLT is non-invasive and generally well-tolerated, its efficacy is still debated, and long-term outcomes remain unclear [48].

Platelet-rich plasma (PRP) therapy involves extracting platelets from the patient’s blood and injecting them into the scalp to stimulate hair growth. Although PRP shows promise, evidence supporting its efficacy is limited, and optimal treatment protocols have yet to be established [49].

The treatment options for hair loss have limitations. These include inconsistent effectiveness among individuals, possible adverse effects, the need for ongoing care, and the high costs associated with certain procedures. Furthermore, it is important to note that not all therapies are universally applicable to all types of hair loss, and the results may differ depending on variables such as age, underlying health conditions, and individual reactions to therapy. It is crucial to conduct future research to uncover new therapeutic targets and improve treatment methods to overcome these constraints and enhance outcomes for individuals experiencing hair loss [11,12,33].

## Nanostructured lipid carriers

**Figure 4.**
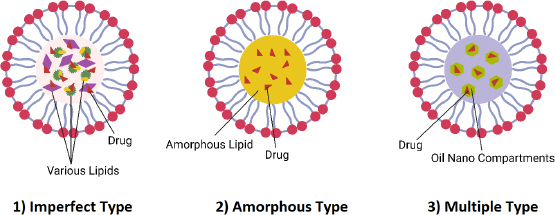
Different NLC types.

NLCs are colloidal drug delivery systems composed of a blend of solid and liquid lipids, often stabilized with surfactants. The unique composition of NLCs imparts several advantageous properties that make them promising candidates for drug delivery applications [50]. The different types of NLC are summarized in Figure 4. Firstly, the imperfect type of NLCs are nanoparticles with an imperfect crystalline lattice structure that contains defects caused by liquid lipids or additives. These imperfections enhance the drug loading capacity and encapsulation efficiency, making them perfect for loading hydrophobic drugs that may not easily blend into highly structured lipid matrices. Secondly, the amorphous type of NLCs have a disordered structure because their lipid matrix lacks long-range order. This structure provides improved drug solubility, higher loading capacity, and enhanced stability. They are ideal for encapsulating hydrophilic drugs or drugs with low aqueous solubility, as they prevent drug expulsion and recrystallization. Finally, the Multiple type of NLCs are nanoparticles containing various lipid components or additives designed to achieve specific properties or functionalities. These can modulate drug release kinetics, improve stability, enhance biocompatibility, or facilitate targeted drug delivery. They offer versatility in formulation design, allowing customization based on drug properties and therapeutic outcomes.

### Composition

Solid lipids (Table 2) form the matrix of NLCs and provide structural integrity to the nanoparticles. These lipids are typically semi-crystalline at room temperature, allowing for the incorporation of lipophilic drugs and enhancing the stability of the formulation [50]. Common solid lipids used in NLCs include glycerides (*e.g.* triglycerides, diglycerides), fatty acids (*e.g.* stearic acid, palmitic acid), and waxes (*e.g.* beeswax, acetyl palmitate) [51].

**Table 2.** Most used solid lipids for NLC composition.

Name	Melting point. °C	*HLB	Formula	Chemical Structure	Ref.
Glyceryl behenate (Compritol^®^ 888 ATO)	65 to 77	2	C_25_H_50_O_4_	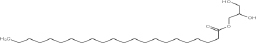	[56]
Cetyl palmitate	54	10	C_32_H_64_O_2_	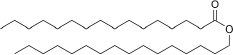	[57]
Stearic acid	69.3	18-20	C_18_H_36_O_2_	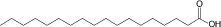	[58]
Glyceryl palmitostearate (Precirol^®^ ATO 5)	52 to 55	2	C_37_H_76_O_7_	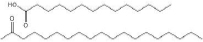	[59]
Cetyl alcohol	33.5 to 35.5	15.5	C_16_H_34_O	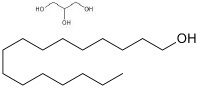	[60]

*HLB refers to the hydrophilic-lipophilic balance

Liquid lipids (Table 3) are incorporated into the lipid matrix to increase the flexibility and fluidity of NLCs. These lipids may be oils or fatty acid esters with low melting points, such as medium-chain triglycerides, vegetable oils, or synthetic esters [52]. Liquid lipids help prevent the complete crystallization of solid lipids, thereby creating nanostructures with higher drug-loading capacity and improved drug-release kinetics [53].

**Table 3.** Most used liquid lipids for NLC composition.

Name	*HLB	Viscosity at 25 °C, cP	Formula	Chemical Structure	Ref.
Caprylic/capric triglycerides (MCTs)	12.5-14	5-20	C_8_H_16_O	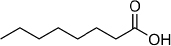	[64]
Isopropyl myristate (IPM)	11.5	3-10	C_17_H_34_O_2_	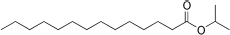	[65]
Ethyl oleate	10.6-11.5	2-5	C_20_H_38_O_2_	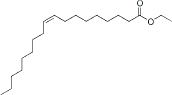	[66]
Coconut oil	8	14-16	C_19_H_21_NO_5_	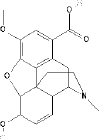	[67]
Squalene	-	3-5	C_30_H_62_	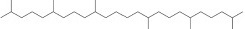	[68]

*HLB refers to Hydrophilic-lipophilic balance

Surfactants (Table 4) are amphiphilic molecules added to NLC formulations to stabilize the nanoparticles and prevent aggregation. These surfactants reduce the interfacial tension between lipid and aqueous phases, facilitating the formation of homogeneous NLC dispersions. Common surfactants include nonionic surfactants like polysorbates (*e.g*. Tween 80), polyethylene glycols (*e.g*., Pluronics), and phospholipids (*e.g*., lecithin) [54,55].

**Table 4.** Most used surfactants for NLC composition.

Name	*HLB	Type	Formula	Chemical Structure	Reference
Tween 80 (Polysorbate 80)	15	Hydrophilic nonionic surfactant	C_64_H_124_O_26_	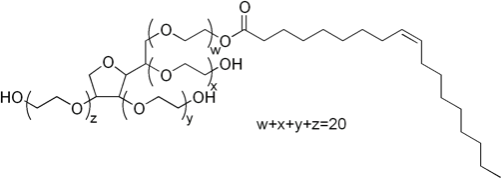	[70]
Span 80 (Sorbitan monooleate)	4.3	Hydrophobic nonionic surfactant	C_24_H_44_O_6_	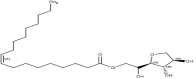	[71]
Poloxamer 188 (Pluronic F68)	29	Amphiphilic nonionic surfactant	C_5_H_14_O_4_	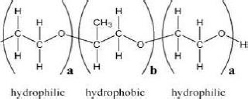	[72]
Lecithin	3-5	Amphiphilic zwitterionic surfactant	C_42_H_80_NO_8_P	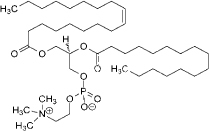	[73]
Polyvinyl alcohol (PVA)	18	Amphiphilic stabilizer	(C_2_H_4_O) _x_	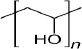	[74]

*HLB refers to hydrophilic-lipophilic balance

### Preparation methods

The preparation of NLCs involves several energy-based methods, each tailored to achieve specific particle characteristics and optimize performance for diverse applications. The common methods are high-pressure homogenization, solvent emulsification-evaporation, microemulsion and coacervation [51].

### Properties

High drug-loading capacity: the unique nanostructure of NLCs allows for the encapsulation of lipophilic and hydrophilic drugs within the lipid matrix, leading to high drug-loading capacities. This property is advantageous for delivering low-solubility or stability therapeutic agents in aqueous media [61].

Controlled drug release, the release pattern of NLCs is a crucial element of their design and operation, impacting the effectiveness and safety of the drugs they deliver. NLCs are designed to offer regulated and prolonged release of the enclosed medication, leading to benefits in maintaining effective drug concentrations and decreasing the need for frequent dosing. The composition of the lipid matrix, the type of drug being encapsulated, and the formulation method all play a role in determining the drug release pattern from NLCs. Changing the composition and structure of the lipid matrix allows for the customization of drug release kinetics in NLCs. Solid and liquid lipids within nanoparticles create a variety of microenvironments that impact the rate of drug diffusion and release. Moreover, surface modifications or coatings can further alter drug release profiles. [62].

Enhanced stability, the lipid-based composition of NLCs imparts excellent stability to encapsulated drugs, protecting them from degradation, enzymatic metabolism, and premature release. Furthermore, the presence of surfactants stabilizes the nanoparticles against aggregation and sedimentation, ensuring uniform dispersion and prolonged shelf-life [63]. Biocompatibility and biodegradability, lipids used in NLC formulations are generally biocompatible and biodegradable, minimizing the risk of toxicity and immunogenicity. This property is particularly important for parenteral administration and long-term drug delivery applications [69]. Versatile formulation flexibility, NLCs offer flexibility in formulation design, allowing for customization of particle size, surface charge, and drug release kinetics to suit specific therapeutic requirements. This versatility enables the development of NLC-based formulations for various routes of administration, including oral, topical, transdermal, and parenteral delivery [75,76].

Overall, NLCs represent a versatile and promising platform for drug delivery, offering controlled release, enhanced stability, and biocompatibility for a wide range of therapeutic applications. Continued research and optimization of NLC formulations hold great potential for advancing drug delivery technologies and improving therapeutic outcomes. NLCs hold significant potential in overcoming the limitations of existing treatments for hair loss by offering targeted and controlled delivery of therapeutic agents to the hair follicles (Table 5) [77].

**Table 5.** Different strategies for overcoming the limitations of hair growth treatments.

Name	Explanation	Effect	Ref.
Dual drug delivery	Complementary drug combination Incorporating multiple drugs. Combining complementary action mechanisms.	Enhancing therapeutic efficacy Overcoming drug resistance.	[90,91]
Targeted delivery	Surface modification of NLCs Utilizing ligands, antibodies, or peptides.	Targeted delivery facilitation Reduces off-target effects. Targets specific cells/tissues.	[92,93]
Therapeutic and imaging agents	Combining therapeutic and imaging agents Fluorescent dyes or contrast agents.	Real-time drug distribution and efficacy monitoring	[94-96]
Sequential release of drugs	Drug release sequence Releases drugs at specific intervals. Response to pH, temperature, and enzymatic activity.	Optimizing drug synergy Minimizing drug interactions. Improving therapeutic outcomes. Delivering drugs at optimal time and concentration.	[97,98]
Gene and drug co-delivery	Co-delivery of therapeutic genes Utilizing siRNA, mRNA, plasmid DNA.	Targeting hair follicle regeneration pathways	[99-101]
Immunotherapy combinations	Promoting hair regrowth Targeting inflammatory processes.	Suppressing autoimmune responses and follicle proliferation	[102,103]
Personalized medicine	Tailoring combination therapy to individual patient profiles Identifying genetic markers or disease characteristics.	Optimizing treatment Minimize adverse effects.	[104,105]

Enhanced drug penetration, one of the limitations of topical treatments for hair loss, such as minoxidil, is limited penetration of the active drug molecules into the deeper layers of the scalp where the hair follicles reside. NLCs can encapsulate these drugs and facilitate their penetration into the hair follicles, enhancing their efficacy [78]. The nanostructured nature of NLCs allows them to bypass the skin barrier and deliver drugs directly to the hair follicles, ensuring more effective treatment of conditions like androgenic alopecia [79].

Sustained release profile, unlike conventional topical formulations, which may require frequent application to maintain therapeutic levels of the drug, NLCs offer controlled release kinetics. This sustained release profile ensures prolonged exposure of the hair follicles to the active drug, potentially reducing the frequency of administration and improving patient compliance [80]. By modulating the lipid composition and surface characteristics of NLCs, the release kinetics can be tailored to match the physiological needs of the hair follicles, optimizing therapeutic outcomes [81].

Follicular targeting, surface modification of NLCs with targeting ligands or peptides can enhance their specificity for hair follicles, minimizing systemic exposure and off-target effects. This targeted delivery approach ensures the hair follicles’ efficient uptake of therapeutic agents, maximizing their bioavailability and minimizing dosage requirements. By selectively targeting the hair follicles, NLCs can mitigate potential side effects of systemic drug exposure, such as hormonal disturbances with oral medications like finasteride [21,82].

Combination therapy, NLCs offer the flexibility to encapsulate multiple therapeutic agents with complementary mechanisms of action in a single formulation. This allows for synergistic combinations of drugs targeting different aspects of hair loss pathology, such as follicular miniaturization, inflammation, and oxidetive stress. Combination therapy delivered via NLCs can potentially enhance treatment efficacy while minimizing the risk of drug resistance and improving patient outcomes [83].

Customization and personalization, NLC formulations can be customized to suit the specific needs of individual patients, considering factors such as disease severity, hair type, and treatment response. This personalized approach ensures optimized therapeutic outcomes and patient satisfaction [84]. Furthermore, NLCs can accommodate a wide range of active pharmaceutical ingredients, including traditional medications, botanical extracts, growth factors, and gene therapies, offering versatility in treatment options for various types of hair loss [85].

In conclusion, NLCs represent a promising strategy for overcoming the limitations of existing treatments for hair loss by providing enhanced drug penetration, sustained release, follicular targeting, combination therapy, and customization. Continued research and development in NLC-based formulations hold great potential for improving the efficacy, safety, and patient acceptance of treatments for hair loss conditions [83,85].

## Surface modification techniques for hair growth treatment

Surface modification techniques are used to enhance the performance of NLCs and play a crucial role in enhancing the performance of NLCs for hair growth treatment by improving their targeting specificity, stability, and drug release characteristics [86]. Several surface modification strategies have been explored to optimize NLC formulations to efficiently deliver therapeutic agents to hair follicles.

### Surface coating

Coating the surface of NLCs with biocompatible polymers, such as polyethylene glycol (PEG) or chitosan, can improve their stability and biocompatibility, prolonging circulation time in biological fluids and reducing immune recognition [87]. Surface coatings also provide steric stabilization, preventing aggregation and opsonization of NLCs, which can enhance their accumulation in hair follicles and improve drug delivery efficiency [11].

### Targeting ligands

Conjugating targeting ligands, such as peptides, antibodies, or aptamers, to the surface of NLCs enables specific binding to receptors or antigens expressed on hair follicle epithelial cells. Targeting ligands enhance the follicular uptake and internalization of NLCs, promoting localized drug delivery to the hair follicles while minimizing off-target effects on surrounding tissues [88]. Examples of targeting ligands include peptides that recognize receptors involved in hair follicle development and growth, such as insulin-like growth factor-1 (IGF-1) receptors or fibroblast growth factor (FGF) receptors [89].

### Cell-penetrating peptides

Cell-penetrating peptides (CPPs) are short peptide sequences capable of facilitating cellular uptake of cargo molecules, including nanoparticles, across biological membranes. Conjugating CPPs to the surface of NLCs enhances their cellular internalization and penetration into the hair follicle epithelium, improving drug delivery efficiency [106,107]. CPPs can also enhance the intracellular trafficking of NLCs, promoting drug release within hair follicle cells and enhancing therapeutic efficacy [108].

### pH-responsive coatings

pH-responsive coatings on NLCs enable triggered drug release in response to changes in the local microenvironment, such as pH variations within the hair follicle [109]. pH-responsive polymers, such as poly acrylic acid or polyethylene glycol, undergo conformational changes in acidic conditions, leading to a controlled release of encapsulated drugs [110]. pH-responsive coatings enhance drug release specificity and reduce systemic exposure, minimizing potential side effects and improving therapeutic outcomes [111].

### Exosome-mimetic coatings

Exosome-mimetic coatings on NLCs utilize cell-derived membrane vesicles as surface modifiers to enhance their biocompatibility and targeting efficiency [112]. Exosome-mimetic coatings mimic the surface properties of natural exosomes, facilitating cellular uptake and intracellular trafficking of NLCs within hair follicle cells. This surface modification strategy improves the bio-interface interactions of NLCs with hair follicle epithelial cells, enhancing their therapeutic efficacy for hair growth treatment [113,114].

Overall, surface modification techniques offer versatile strategies for enhancing the performance of NLCs for hair growth treatment, enabling targeted and efficient delivery of therapeutic agents to the hair follicles. Continued research and innovation in this field hold great promise for developing advanced NLC formulations with improved efficacy, safety, and patient acceptance.

### The importance of surface modifications

Surface modification is pivotal in improving drug delivery systems’ targeting, penetration, and efficacy, including NLCs. By modifying the surface properties of NLCs, it becomes possible to enhance their interactions with biological targets, optimize their biodistribution, and promote efficient drug delivery to specific sites of action [115].

Enhanced targeting of NLCs and surface modification allows for the attachment of targeting ligands, such as peptides, antibodies, and aptamers. These ligands can specifically recognize and bind to receptors or antigens overexpressed on the target cells or tissues, facilitating targeted delivery [116]. By directing NLCs to the desired site of action, targeting ligands increases the accumulation of therapeutic agents within the target cells or tissues while minimizing off-target effects on healthy tissues. Targeted delivery enhances the efficacy of NLC-based therapies and reduces the required dosage, potentially mitigating systemic side effects and improving patient safety [117].

A higher penetration rate by surface modification can improve NLC penetration across biological barriers, such as the blood-brain barrier and the skin’s stratum corneum. Coating NLCs with CPPs facilitates cellular internalization and intracellular trafficking, enabling efficient penetration into target cells and tissues. pH-responsive coatings on NLCs can promote triggered drug release in response to changes in the local microenvironment, facilitating deeper penetration into target tissues, such as acidic tumour microenvironments or inflamed skin [108].

Optimum biodistribution by surface modification influences NLC biodistribution in the systemic circulation by modulating their interaction with biological components. Coating NLCs with biocompatible polymers, such as polyethylene glycol (PEG), reduces nonspecific protein adsorption and opsonization, prolonging their circulation time and enhancing their accumulation at the target site [118]. Stealth properties conferred by surface modification with PEG or other hydrophilic polymers minimize recognition by the reticuloendothelial system (RES), reducing clearance by phagocytic cells and improving the availability of NLCs for targeted drug delivery [119].

Enhanced efficacy by Surface modification optimizes drug delivery parameters, including release kinetics, localization, and duration of action, in NLC-based therapies. By enhancing targeting specificity and penetration into target cells or tissues, surface-optimized NLCs ensure efficient delivery of therapeutic agents to the desired site of action, maximizing their pharmacological effects [120]. Controlled drug release from pH-responsive or stimuli-responsive coatings further enhances therapeutic efficacy by providing spatiotemporal control over drug release, minimizing premature release, and optimizing drug availability at the target site [121].

In summary, surface modification of NLCs is instrumental in improving drug delivery systems’ targeting, penetration, and efficacy. By fine-tuning the surface properties of NLCs, it becomes possible to achieve precise control over drug delivery parameters, enhance therapeutic outcomes, and minimize off-target effects, ultimately advancing the field of targeted drug delivery for various biomedical applications.

## Commonly used surface modification methods

**Figure 5.**
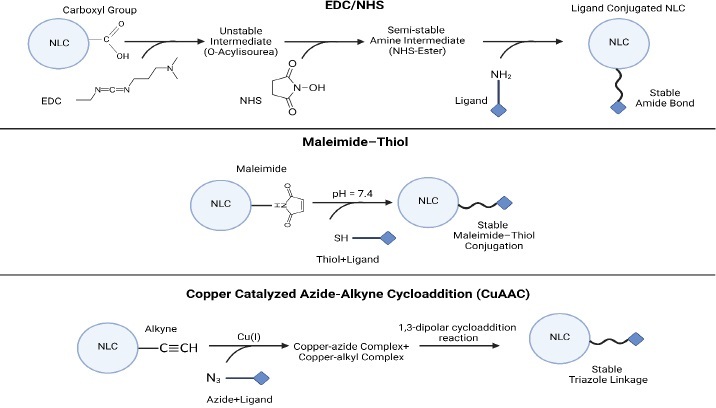
Covalent surface activation techniques.

**Figure 6.**
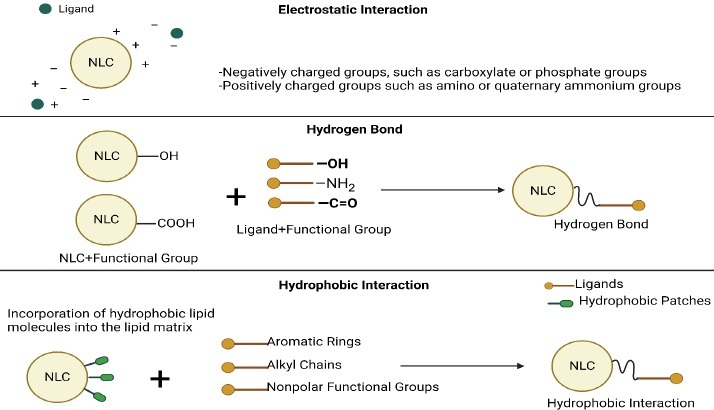
Non-Covalent surface activation techniques.

Common surface modification methods for NLCs include ligand conjugation, polymer coating, and peptide functionalization (Table 6). These methods enable the customization of NLCs for specific therapeutic applications by imparting desired surface properties and functionalities [69]. Some covalent and non-covalent techniques for activating surfaces for modifications should be applied in advance (Figures 5 and 6).

**Table 6.** Common surface modification methods.

Modification technique	Advantages	Disadvantages	Ref.
Ligand conjugation	Enhanced targeting specificity Reduced off targets effects Improved pharmacokinetics Tailored drug delivery	Complexity of conjugation Loss of ligand activity Batch-to-batch variability Non-specific binding Immune response Stability Issues	[116,122,123]
Polymer coating	Enhanced stability Controlled drug release Iimproved prolonged circulation Improved pharmacokinetics Scalability and reproducibility	Complexity of formulation Reduction in drug loading capacity Potential for drug-polymer interactions Alteration of surface properties Stability issues	[124,125]
Peptide functionalization	Enhanced targeting specificity Biological compatibility Diverse targeting options Controlled drug release Multifunctionality Stability enhancement Reduced clearance Versatility in formulation	Complexity of peptide synthesis Potential immunogenicity Specificity and affinity toward ligand Batch-to-batch variability Limited cargo capacity Cellular uptake limitations	[126,127]

### Ligand conjugation

Principle: Ligand conjugation involves attaching targeting ligands, such as peptides, antibodies, or aptamers, to the surface of NLCs via chemical or physical interactions [116].

Procedure: Functionalization of NLC surface with linker molecules, such as polyethylene glycol (PEG) or other reactive groups, to create sites for ligand attachment [128]. A targeting ligand is conjugated to a surface-functionalized NLC by covalent bonding or non-covalent interactions, such as hydrophobic interactions or electrostatic interactions [129].

### Polymer coating

Principle: Polymer coating involves encapsulating NLCs within a layer of biocompatible polymers, such as polyethylene glycol (PEG), chitosan, or poloxamers, to modify their surface properties and enhance stability [87].

Procedure: NLCs are encapsulated within a polymer solution, typically by emulsion-based or nanoprecipitation techniques [130]. Also, the polymer solution is added to the NLC dispersion, forming a coating around the NLCs through self-assembly or solvent evaporation [87].

### Peptide functionalization

Principle: Peptide functionalization involves attaching CPPs or other bioactive peptides to the surface of NLCs to enhance cellular uptake and intracellular delivery of therapeutic agents [108].

Procedure: NLCs are modified with linker molecules, such as maleimide-functionalized lipids or cross-linkers, to introduce reactive groups for peptide conjugation [131]. Also, peptides containing reactive groups, such as cysteine residues or amino groups, are conjugated to the surface-modified NLCs through covalent bonding [132].

Therefore, surface modification methods such as ligand conjugation, polymer coating, and peptide functionalization offer versatile strategies for customizing the surface properties and functionalities of NLCs for targeted drug delivery applications. By selecting appropriate surface modification techniques, it becomes possible to optimize the performance of NLCs and tailor them for specific therapeutic indications, enhancing their efficacy and clinical utility [116].

## Application of surface-modified NLCs

Surface-altered NLCs hold huge potential across various pharmaceutical applications, cosmetics, and personal care products, offering efficient and targeted delivery of therapeutic and cosmetic agents to the skin and hair [133]. Continued research and development in this field are essential for finding new opportunities and addressing emerging challenges in drug delivery studies. Recent studies are summarized in Table 7.

**Table 7.** NLCs based on surface modifications.

No.	Drug	Target	Surface modification	Technique	Ref.
1	Quercetin	Gastrointestinal	Alginate hydrogel	Ionic gelation process	[136]
2	Tyrosine kinase Inhibitor	Hepatocellular Carcinoma	Pentapeptide cRGDfK	Carbodiimide reaction	[137]
3	Gambogic acid	MDA-MB-231	Two kinds of cell-penetrating peptides (cRGD and RGERPPR)	EDC/NHS	[138]
4	Docetaxel	Hepsin (Hpn)-expressing cancer cells	RIPL peptide	Thiol-maleimide reaction	[139]
5	Tranylcypromine	Cell lines Caco-2 and SH-SY5Y	Model amphipathic peptide	Click chemistry (CuAAC)	[140]
6	-	Caco-2 cells	Oleoyl-quaternized-chitosan	Electrostatic interactions	[141]
7	Tamoxifen	Cancer cells	Polyoxyethylene (40) stearate	Hydrophilic bond	[142]
8	Metformin	Diabetes treatment	PEG	Polymer coating	[143]
9	Dexamethasone acetate	Eye	Polymyxin B sulfate	N-acetylcysteine-functionalized	[144]
10	Donepezil	Brain	Chitosan-coated	Polymer coating	[145]

**Figure 7.**
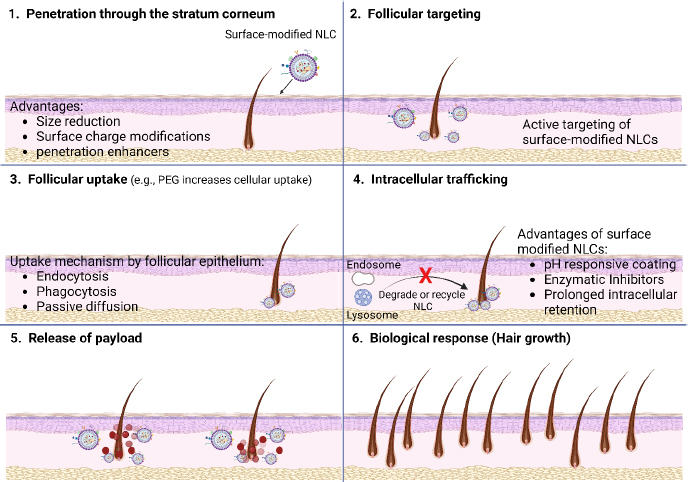
Follicular delivery of surface-modified NLCs.

Surface-functionalized NLCs hold promise for stimulating hair follicle growth and reducing hair loss through multiple mechanisms of action. [134]. These mechanisms involve targeted delivery of bioactive molecules to the hair follicles, modulation of signalling pathways involved in hair growth and follicle cycling, and enhancement of follicular microenvironment (Figure 7) [135]. Figure 7(1) illustrates that the surface-optimized NLCs utilize their properties, such as size, shape, and surface charge, to navigate through the complex microenvironment of the skin and reach the hair follicles. These nanoparticles can penetrate through the stratum corneum, the outermost layer of the skin, and interact with the follicular epithelium. Figure 7(2) indicates that the follicular targeting of surface-modified NLCs involves many approaches to directing these nanoparticles specifically to the hair follicles and enhancing drug delivery efficacy. Figure 7(3) illustrates that the ligands bind to receptors or proteins expressed on the follicular epithelial cells, promoting the selective uptake of NLCs into the hair follicles. Receptor-mediated endocytosis is a common mechanism by which surface-functionalized NLCs are internalized into follicular cells. Once bound to the follicular epithelium, surface-altered NLCs can be internalized via various pathways, including endocytosis, phagocytosis, or passive diffusion. Further, Figure 7(4) indicates that following endocytosis, surface-modified NLCs undergo intracellular trafficking within the follicular cells. These nanoparticles may be transported through the endosomal-lysosomal pathway, where they encounter acidic and enzymatic environments that can trigger drug release from the NLCs. Alternatively, NLCs may bypass lysosomal degradation and escape into the cytoplasm, leading to prolonged retention within the follicular cells. Furthermore, once inside the follicular cells, surface-modified NLCs release their payload of therapeutic agents (Figure 7(5)) and may exert their pharmacological effects locally within the hair follicles or systemically via absorption into the bloodstream (Figure 7(6)).

Targeted delivery to hair follicles by surface modification of NLCs enables specific binding to receptors or antigens expressed on hair follicle epithelial cells. This facilitates targeted delivery of therapeutic agents to hair follicles. Ligand-conjugated NLCs can selectively bind to receptors such as insulin-like growth factor-1 (IGF-1) receptors, fibroblast growth factor (FGF) receptors, or vascular endothelial growth factor (VEGF) receptors, which are known to play crucial roles in hair follicle development, angiogenesis, and regulation of the hair growth cycle. By delivering growth factors, cytokines, or small interfering RNA (siRNA) directly to the hair follicles, surface-modified NLCs can modulate signaling pathways involved in hair follicle growth, proliferation, and differentiation [146,147].

Activation of hair follicle stem cells by surface-modified NLCs loaded with growth factors or bioactive peptides can stimulate hair follicle stem cells (HFSCs), which reside in the bulge region of hair follicles and play a key role in hair regeneration and cycling. Growth factors such as FGFs, VEGF, and insulin-like growth factor (IGF) promote the activation and proliferation of HFSCs, leading to increased follicular regeneration and hair growth. By delivering growth factors directly to the hair follicles, surface-altered NLCs can enhance the activation and function of HFSCs, promoting hair follicle growth and regeneration [148-150].

Modulation of inflammatory responses like Inflammatory cytokines and immune-mediated processes play a significant role in the pathogenesis of hair loss disorders such as alopecia areata and telogen effluvium. Surface-optimized NLCs loaded with anti-inflammatory agents or immunomodulatory drugs can attenuate inflammatory responses in the hair follicles, reducing follicular damage and promoting hair growth [151]. By delivering anti-inflammatory agents such as corticosteroids or immunosuppressants directly to the hair follicles, surface-modified NLCs can suppress inflammatory cytokine production and inhibit immune cell infiltration, thereby preventing follicular miniaturization and hair loss [152].

Enhancement of follicular microenvironment by Surface-modified NLCs can create a favorable microenvironment within the hair follicles by delivering growth factors, antioxidants, or nutrients that support follicular growth and maintenance. Growth factors such as VEGF and FGF promote angiogenesis and improve blood supply to the hair follicles, ensuring adequate nutrient and oxygen delivery for follicular growth. Antioxidants such as vitamins and polyphenols protect hair follicles from oxidative stress and free radical damage, preserving follicular integrity and promoting hair growth [153,154].

In summary, surface-activated NLCs affect hair follicle growth and hair loss reduction through targeted delivery of bioactive molecules, activation of hair follicle stem cells, modulation of inflammatory responses, and enhancement of the follicular microenvironment. These mechanisms collectively promote hair growth, follicular regeneration, and maintaining healthy hair follicles, highlighting the potential of surface-modified NLCs as innovative therapeutic strategies for hair loss disorders.

Several bioactive compounds and growth factors (Table 8) have been encapsulated within surface-activated NLCs for hair growth treatment. These encapsulated agents target various aspects of hair follicle physiology and can promote hair growth by stimulating follicular proliferation, angiogenesis, and regulation of the hair growth cycle [134]. Table 8 shows examples of specific bioactive compounds and growth factors incorporated into surface-modified NLCs for hair growth treatment.

**Table 8.** Examples of specific bioactive compounds or growth factors encapsulated within surface-modified NLCs for hair growth treatment.

Effective ingredient	Modification technique	Effects	Ref.
Minoxidil	Polymer coating Peptide Functionalization	Promoting hair growth Stimulating blood flow to the hair follicles Prolonging the anagen (growth) phase of the hair growth cycle	[156]
Growth factors	Polymer coating Ligand conjugation	Promoting angiogenesis Stimulating hair follicle stem cell proliferation Regulating the hair growth cycle	[157]
Peptides	Ligand conjugation Peptide Functionalization	Therapeutic effects Targeted delivery Prolong release kinetics	[158]
Natural extracts	Polymer coating Ligand conjugation	Hair growth-promoting Promotion of dermal papilla cell proliferation Modulation of inflammatory responses	[135]

These examples highlight the versatility of surface-activated NLCs as drug delivery vehicles for encapsulating a wide range of bioactive compounds and growth factors for hair growth treatment. By optimizing formulation parameters and surface modification strategies, it is possible to develop NLC-based formulations with enhanced efficacy and targeted delivery for the management of hair loss disorders.

## Future perspectives and challenges

The potential future developments and applications of surface-altered NLCs in hair growth treatment are vast and promising, offering innovative solutions to address the challenges associated with current therapies for hair loss disorders [155].

Advances in surface modification techniques, such as ligand conjugation and peptide functionalization, enable the customization of NLC formulations for individual patients based on their specific hair loss conditions, genetic factors, and treatment responses [159]. Tailored NLC formulations can be designed to target specific molecular pathways implicated in different types of hair loss disorders, allowing for precision medicine approaches that optimize therapeutic outcomes and minimize side effects [21].

Combination therapy and synergistic formulations by surface-modified NLCs offer opportunities for combining multiple therapeutic agents with complementary mechanisms of action in a single formulation. Combination therapy with NLCs can synergistically target various pathways involved in hair follicle growth and regeneration, such as angiogenesis, stem cell activation, and inflammation modulation, leading to enhanced efficacy and improved treatment outcomes [55].

Gene therapy and RNA-based therapeutics with surface-altered NLCs can serve as effective carriers for delivering gene therapy vectors or RNA-based therapeutics, such as small interfering RNA (siRNA) or microRNA (miRNA), to modulate gene expression patterns associated with hair growth and follicle cycling [160]. Gene therapy approaches delivered via NLCs hold promise for targeting specific genes involved in hair follicle development, differentiation, and cycling, offering potential long-term solutions for hereditary hair loss disorders [161].

Stem cell-based therapies and tissue engineering and Surface-altered NLCs can deliver growth factors, cytokines, and signaling molecules that promote stem cell proliferation, differentiation, and engraftment within the hair follicles. Stem cell-based therapies combined with NLC-mediated delivery of bioactive factors can regenerate damaged or dormant hair follicles, restoring hair growth in conditions such as androgenic alopecia and scarring alopecia [162].

Nanotechnology-based imaging and diagnostics of surface-altered NLCs can be engineered to incorporate imaging or contrast agents for non-invasive imaging and diagnostic applications related to hair follicle health and function [150]. Nanotechnology-based imaging techniques, such as nanoparticle-enhanced imaging or fluorescence microscopy, can provide insights into hair follicle morphology, blood flow, and cellular dynamics, aiding in diagnosing and monitoring hair loss disorders [163].

Smart drug delivery systems and stimulus-responsive formulations with future developments in surface-modified NLCs may involve designing smart drug delivery systems capable of responding to specific stimuli or environmental cues within the hair follicle microenvironment. Stimulus-responsive NLC formulations, such as pH-responsive or temperature-sensitive systems, can provide controlled and on-demand release of therapeutic agents within the hair follicles, optimizing treatment efficacy and minimizing systemic side effects [147,164].

Overall, the future of surface-modified NLCs in hair growth treatment holds great promise for advancing therapeutic strategies, enhancing treatment efficacy, and improving patient outcomes in managing hair loss disorders. Continued research and innovation in this field are essential for realizing the full potential of NLC-based formulations as next-generation therapies for hair growth promotion and restoration.

## The challenges for surface-modified NLC-based hair growth therapies

Although NLCs have the potential to serve as innovative platforms for hair growth treatments, their practical application in clinical translation, capacity to be scaled up, and meeting regulatory requirements present significant obstacles [150].

Efficacy validation: Clinical translation of NLC-based hair growth therapies requires rigorous validation of efficacy, safety, and tolerability in human clinical trials. Designing well-controlled studies with appropriate endpoints and patient populations is essential to demonstrate the therapeutic benefits of NLC formulations [165].

Long-term safety: Ensuring the long-term safety and stability of NLC formulations is crucial for clinical translation. Comprehensive toxicological studies and pharmacokinetic assessments are necessary to evaluate potential adverse effects and ensure patient safety during prolonged treatment [166].

Production scale-up: Scaling up the production of NLC formulations to meet commercial demand poses challenges in maintaining batch-to-batch consistency, optimizing manufacturing processes, and ensuring product quality and stability [167].

Cost considerations: The cost of manufacturing NLC formulations at scale may be higher than conventional formulations, primarily due to the complexity of nanoparticle production processes and the need for specialized equipment and expertise [168].

Regulatory requirements: NLC-based hair growth therapies must undergo rigorous regulatory review and approval by health authorities, such as the U.S. Food and Drug Administration (FDA) or the European Medicines Agency (EMA). Meeting regulatory requirements for safety, efficacy, and quality control is time-consuming and resource-intensive [169].

Quality control standards: Establishing robust quality control standards for NLC formulations is essential to ensure consistency, purity, and stability throughout the manufacturing process and product lifecycle. Compliance with Good Manufacturing Practice (GMP) guidelines is mandatory for regulatory approval [168,170].

Patent protection: Securing patent protection or intellectual property rights for NLCs is crucial for fostering innovation, guaranteeing economic profits, maintaining product integrity and safety, enhancing public health, and encouraging knowledge exchange. Patents give researchers and companies the motivation and protection to invest in and advance cutting-edge NLC technologies, which ultimately help society. Patent protection may be challenging due to the complex and multidisciplinary nature of nanoparticle formulations and surface modification techniques [171].

Market competition: The hair growth market is highly competitive, with numerous established and emerging treatments available, including topical solutions, oral medications, and invasive procedures. Differentiating NLC-based therapies from existing treatments and gaining market acceptance may be challenging [172].

Treatment accessibility: Ensuring accessibility and affordability of NLC-based hair growth therapies for patients from diverse socioeconomic backgrounds is important to maximize patient access and adherence to treatment regimens [134].

User-friendly formulations: Developing user-friendly, easy-to-apply, non-greasy, and cosmetically acceptable formulations can improve patient acceptance and compliance with NLC-based hair growth treatments [173]. Addressing these challenges will require collaborative efforts from researchers, industry partners, regulatory agencies, and healthcare providers to advance the clinical development and comercialization of NLC-based hair growth therapies. Overcoming these challenges has the potential to unlock the therapeutic benefits of NLC formulations and improve outcomes for patients with hair loss disorders [174].

## Conclusions

The potential to revolutionize hair growth treatment lies in the surface modification of NLCs, which can enhance the targeted transport of therapeutic medicines to hair follicles. Nanoparticle lipid carriers NLCs can enhance the effectiveness of drug administration by selectively binding to receptors or antigens expressed on hair follicle cells. This is achieved using ligand conjugation, polymer coating, and peptide functionalization techniques. This enables accurate administration of bioactive chemicals, growth factors, and gene therapies to the hair follicles, improving their therapeutic effectiveness in boosting hair growth, promoting follicular regeneration, and minimizing hair loss. Surface modification enables the customization of NLC formulations to specifically target the pathways involved in hair follicle physiology and growth control. This provides personalized therapy options for various hair loss problems and patient requirements. NLCs coated with polymers or functionalized with peptides can release therapeutic medicines regulated within the hair follicles. This allows for a longer duration of action and reduces adverse effects throughout the body. Surface-activated NLCs can combine various therapeutic drugs with complementary modes of action in a single formulation. This allows for synergistic effects and enhanced treatment results through combination therapy approaches. Although NLC-based hair growth therapies have the potential to be beneficial, their clinical translation and regulatory approval are hindered by problems with the validation of efficacy, scalability, manufacturing, and regulatory compliance. To overcome current obstacles and successfully implement hair growth treatments that are safe, effective, and patient-centered, it is crucial to do additional research and foster collaboration among academics, industry, healthcare providers, and regulatory bodies. To summarize, the alteration of the surface of NLCs shows potential for revolutionizing hair growth treatment by providing precise and effective transportation of medicinal substances to hair follicles. Additional research, innovation, and collaboration are necessary to enhance treatment outcomes and tackle current obstacles.
